# Entomological Risk Assessment for Dengue Virus Transmission during 2016–2020 in Kamphaeng Phet, Thailand

**DOI:** 10.3390/pathogens10101234

**Published:** 2021-09-24

**Authors:** Thanyalak Fansiri, Darunee Buddhari, Nattaphol Pathawong, Arissara Pongsiri, Chonticha Klungthong, Sopon Iamsirithaworn, Anthony R. Jones, Stefan Fernandez, Anon Srikiatkhachorn, Alan L. Rothman, Kathryn B. Anderson, Stephen J. Thomas, Timothy P. Endy, Alongkot Ponlawat

**Affiliations:** 1 Department of Entomology, Armed Forces Research Institute of Medical Sciences (AFRIMS), Bangkok 10400, Thailand; ThanyalakF.fsn@afrims.org (T.F.); NattapholP.ca@afrims.org (N.P.); ArissaraP.ca@afrims.org (A.P.); 2 Department of Virology, Armed Forces Research Institute of Medical Sciences (AFRIMS), Bangkok 10400, Thailand; DaruneeT.fsn@afrims.org (D.B.); ChontichaK.fsn@afrims.org (C.K.); anthony.jones.mil@afrims.org (A.R.J.); stefan.fernandez.mil@afrims.org (S.F.); 3 Department of Disease Control, Ministry of Public Health, Nonthaburi 11000, Thailand; sopon@ddc.mail.go.th; 4 Faculty of Medicine, King Mongkut’s Institute of Technology Ladkrabang, Bangkok 10520, Thailand; anons.gst@afrims.org; 5 Department of Cell and Molecular Biology, Institute for Immunology and Informatics, University of Rhode Island, Providence, RI 02903, USA; alan_rothman@uri.edu; 6 Department of Medicine, SUNY Upstate Medical University, Syracuse, NY 13210, USA; AndeKath@upstate.edu (K.B.A.); ThomStep@upstate.edu (S.J.T.); Endyt@upstate.edu (T.P.E.)

**Keywords:** *Aedes aegypti*, *Aedes albopictus*, mosquito surveillance, dengue, Kamphaeng Phet, Thailand

## Abstract

Individual houses with high risks of dengue virus (DENV) transmission might be a source of virus transmission within the neighborhood. We conducted an entomological risk assessment for DENV transmission at the household level, comprising family cohort members residing in the same location, to assess the risk for dengue virus transmitted by mosquito vectors. The studies were conducted in Kamphaeng Phet Province, Thailand, during 2016–2020. Entomological investigations were performed in 35 cohort families on day 1 and day 14 after receiving dengue case reports. DENV was found in 22 *Aedes* samples (4.9%) out of 451 tested samples. A significantly higher DENV infection rate was detected in vectors collected on day 1 (6.64%) compared to those collected on day 14 (1.82%). Annual vector surveillance was carried out in 732 houses, with 1002 traps catching 3653 *Aedes* females. The majority of the 13,228 water containers examined were made from plastic and clay, with used tires serving as a primary container, with 59.55% larval abundance. Larval indices, as indicators of dengue epidemics and to evaluate disease and vector control approaches, were calculated. As a result, high values of larval indices indicated the considerably high risk of dengue transmission in these communities.

## 1. Introduction

Dengue fever is one of the most serious public health threats to humans [1]. The epidemiology and disease burden have been described in the regions of South-East Asia, the Western Pacific, Africa, the Americas, and the Eastern Mediterranean [2]. The frequency and magnitude of dengue epidemics have increased dramatically as dengue virus (DENV) and the mosquito vectors have both expanded geographically in tropical and subtropical regions throughout the world [3]; as a result, more than 3.9 billion people in over 129 countries are at risk, with an estimated 96 million symptomatic cases and an estimated 40,000 deaths each year [4]. DENV is found in almost every urban and peri-urban area in the tropics and subtropics where mosquito vectors exist. Thailand is highly hyperendemic for dengue and suffers from one of the highest rates of dengue in the world. In 2020, Thailand's Ministry of Public Health (MOPH) reported 50,670 (DF), 20,908 (DHF), and 552 (DSS) cases across all provinces [5].

DENV-1, DENV-2, DENV-3, and DENV-4 are four closely related serotypes that cause different illnesses, including dengue fever (DF), dengue hemorrhagic fever (DHF), and dengue shock syndrome (DSS) [6]. No vaccine is available and the routine use of insecticides as part of the vector control program has been ineffective, resulting in resistance in *Aedes aegypti* and *Ae. albopictus* in dengue-endemic areas [7,8,9]. As a consequence, dengue fever remains an important disease throughout the tropics, with the potential to further expand geographically. This rapid spread of viral infection could possibly be caused by a combination of factors such as the massive susceptible population, climatic conditions that are suitable for mosquito vector development, other possibilities of non-vector transmission, and a high rate of population movement [10]. Travelers from regions where arbovirus transmission is prevalent play a critical role in the spread of these infections, whether they are traveling internationally or domestically. These viremic travelers have the potential to spread viruses to non-endemic countries [2,11]. 

The yellow fever mosquito, *Ae. aegypti*, and the Asian tiger mosquito, *Ae. albopictus*, are major vectors of DENV. These mosquito species are anthropophilic and highly adapted to urban environments due to breeding in water storage containers, garbage, and discarded containers [6,12]. *Aedes* mosquitoes have a wide range of breeding habitats, from natural, such as coconut shells, to man-made, such as water storage containers and discarded tires. They prefer to lay eggs on the inner wet walls of water storage containers. Under dry conditions, the eggs can survive for a long period of dormancy, until after rainfall; when the containers are filled with water, the eggs will be hatched and develop to the next instar [13]. Mosquito vectors become infected when they feed on viremic patients in which there are sufficient circulating viral particles to provide an infectious dose to the vectors [6].

Vector control and prevention can be performed by increasing public awareness and encouraging citizens to take control of mosquito breeding sites around their residences and use repellents and chemical control measures. In Thailand, adulticide spraying and larvicide application are part of the vector control program. Synthetic pyrethroid adulticides including deltamethrin 0.5% emulsifier concentrate formulation (EC) and zeta-cypermethrin 2.25% EC have been routinely used by the local public health officers, who spray adulticides at the dengue index house and houses located within a 100-m radius of the index house within 24 h of each dengue case report. The organophosphate larvicide (temephos) has been widely utilized in Thailand's national *Aedes* larval control program since the 1950s and has historically been highly effective in controlling *Aedes* larvae in most regions of the country [9]. Temephos remains routinely used throughout Thailand due to its low mammalian toxicity, long-lasting effect, and low operational cost. However, since temephos has been routinely used in Thailand for more than 70 years, the resistance of *Ae. aegypti* and *Ae. albopictus* to temephos in many dengue-endemic areas in Thailand has been reported [7,8].

Entomological surveillance against vector infestation is very important in predicting the occurring of disease outbreak [14]. It is applied to determine vector population abundance and vector distribution changes over time and can be used for monitoring and evaluating the effectiveness of vector control programs [2]. This facilitates appropriate and timely decisions regarding disease control interventions. Previous studies conducted by researchers from the Armed Forces Research Institute of Medical Sciences (AFRIMS) have demonstrated spatial and temporal fluctuations in DENV transmission in populations in Kamphaeng Phet (KPP) [15,16,17]. These studies have revealed the important aspect of virus–host interactions either within a single household or in neighboring houses in close proximity. It is extremely important to focus on DENV transmission at the household level. Results from cluster investigations in KPP showed the spatial aggregation of DENV infections and high density of *Ae. aegypti* pupae per person [15]. In dengue-positive cluster investigations in 2012, over 8% of *Ae. aegypti* collected from houses associated with dengue cases were DENV PCR-positive, while only 0.4% collected from houses without dengue cases were DENV PCR-positive [17]. This study reported a positive association between DENV infection in human hosts and mosquito vectors. A remarkably high risk of human DENV infection was found in houses with DENV-infected mosquitoes and with high mosquito population density. Their neighborhoods likewise had a high risk of human infection. The authors revealed that the most important association was at the individual house level. Human and mosquitoes at small geographic and temporal scales were responsible for a larger part of DENV transmission. Since the flight range of major DENV vectors, *Ae. aegypti*, is approximately 50–100 m from the breeding sites [18], other family members living in the same area as the dengue-infected patients can be easily exposed to the infected mosquitoes and have a similar entomological risk. A family cohort study of dengue in households in KPP has been conducted by the AFRIMS Virology Department, aiming to determine the incidence of DENV infection in a prospective longitudinal cohort of family units containing family members of all ages.

To determine the risk of DENV transmission between mosquito vectors and different family members with different pre-exposure histories, we looked at the DENV infection rate in *Aedes* adults from the KPP family cohort study, as well as the infestation of *Aedes* larvae in all water-holding containers that serve as potential breeding sites of dengue vectors in KPP. The findings of this study can be incorporated into dengue preventive and control measures to estimate the risk of DENV transmission in both disease-endemic and non-endemic countries. Furthermore, researchers and operational personnel responsible for managing disease outbreaks or establishing preventive control programs would benefit from the valuable information on larval habitats and mosquito prevalence. 

## 2. Results

### 2.1. Entomological Study in Dengue Case Areas

#### 2.1.1. Adult Mosquito Infestation in Dengue Transmission Areas

*Aedes* adults were collected on both collection dates (day 1 and day 14) from all 51 study households. Adult vectors were found in 82.3% (84/102) of the total examined houses, according to Table 1. On both collection days, a total of 204 BG traps were deployed inside houses. A total of 1216 mosquitoes from five genera (*Aedes*, *Culex*, *Anopheles*, *Mansonia*, and *Armigeres*) were collected. On day 14, the total number of mosquitoes, *Aedes* females, and other mosquito species were significantly lower (n = 475, 181, 294) than on day 1 (n = 741, 311, 430) (Wilcoxon signed rank test: *p* = 0.002, 0.002, 0.019, respectively, Table 1). The collected *Aedes* females (n = 492; 40.5%) were identified as *Ae. aegypti* (n = 488) and *Ae. albopictus* (n = 4), with an average of two *Aedes* females per trap. *Aedes* males, *Culex* spp., *Anopheles* spp., *Mansonia* spp., and *Armigeres* spp. were detected among the remaining mosquitoes (n = 724; 59.5%).

#### 2.1.2. Infection Status of the Collected Mosquitoes 

**Figure 1:**
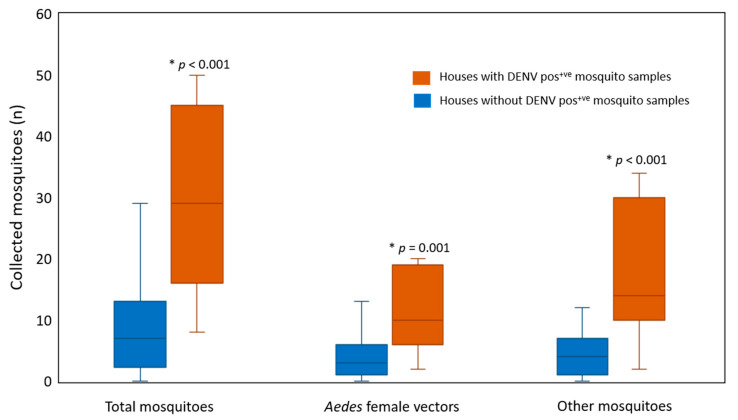
Comparison of the numbers of collected mosquitoes between houses with and without DENV-positive mosquito samples. * *p*-value < 0.05; Pos^+ve^: Positive.

Our results demonstrated that 22 out of 451 mosquitoes were infected with DENV (4.9% infection rate) in the following order: DENV-1 (n = 6), DENV-2 (n = 5), DENV-3 (n = 6), and DENV-4 (n = 5) (Table 1). Differences in the DENV infection rate detected in *Aedes* females collected on day 1 and day 14 were analyzed. A higher DENV infection rate was detected in *Aedes* females collected on day 1 (6.64%) compared to day 14 (1.82%) (Fisher’s exact test: *p* = 0.023) with a concomitant decrease in the infection rate found in vectors collected from index houses (day 1 = 8.57%, day 14 = 2.57%) (Fisher’s exact test: *p* = 0.036) (Table 1). Additionally, DENV infection rates were compared between the vectors collected from the index and neighboring houses. The DENV infection rate in mosquitoes captured in index houses (6.44%) was higher than in neighboring houses (0.8%), indicating that infection status was associated with study house conditions (Fisher’s exact test: *p* = 0.012) (Table 2). Therefore, *Aedes* females in index houses had a high level of DENV infection. Moreover, houses with DENV-infected mosquitoes had higher numbers of total collected mosquitoes, *Aedes* females, and other mosquitoes than neighboring houses (Mann–Whitney U test: *p* < 0.001, *p* = 0.001, *p* < 0.001). (Figure 1). None of the *Ae. albopictus* collected in this study were found to be infected with DENV. In our investigation, most of the DENV serotypes in the tested mosquitoes matched those DENV identified in human index cases. Only two DENV-4-positive mosquito samples were collected in the houses of DENV-1-positive index cases on day 1 and day 14 (index case no. 34, 35; Table S1).

#### 2.1.3. Larval Indices and Breeding Container Availability in Dengue Transmission Areas

*Aedes* larval surveys were performed inside and around the areas of 51 houses on day 1 following dengue case reports. During 2016–2020, all 800 water-holding containers were examined for the presence of *Aedes* larvae (573 and 227 containers in index and neighboring houses, respectively) (Table 3). The presence of *Aedes* larvae was related to study house conditions according to the Chi-square test, with the proportion of positive containers in the index houses (18.67%) being greater than that in the neighboring houses (11.01%; χ^2^ = 6.38, df = 1, *p* = 0.012). Because the presence of larvae was related to the prevalence of DENV-infected mosquitoes in the study households (26.61% vs. 11.96%), significantly more positive containers were detected in houses with DENV-infected mosquitoes (χ^2^ = 25.63, df = 1, *p* < 0.001, Table 3).

### 2.2. Annual Entomological Surveillance 

#### 2.2.1. Adult Mosquito Infestation 

Adult mosquito collections were conducted in 501 houses by using 1002 BG traps to collect 6920 mosquitoes, which were identified as five genera of *Aedes*, *Culex*, *Mansonia*, *Anopheles*, and *Armigeres* (Table 4A). Among these collected samples, 3653 samples were identified as *Aedes* females (*Ae. aegypti* = 3604, *Ae. albopictus* = 49). Based on total five-year collections, the number of *Aedes* vectors in all study areas per year ranged from 221 to 1166 females; in commercial city areas, it ranged from 109 to 791, and in rural areas, it ranged from 112 to 375 (Table 4).

#### 2.2.2. Larval Indices 

The presence of *Aedes* larvae in water-holding containers was investigated both inside and outside the study households, and the numbers of houses inspected in annual entomological surveillance were calculated as larval indices (house index: HI, container index: CI, and Breteau index: BI) presented in Table 5. All larval indices were reported at 95% confidence interval (95% CI). During the years 2016–2020, annual larval surveys were performed in Muang district and Khanu Woralaksaburi district in a total of 732 houses, including all 501 houses where adult mosquito collections were performed (Table 5). Our results demonstrated that 630 houses tested positively for *Aedes* larval presence. A total of 2650 out of 13,228 water-holding containers inspected were positive for *Aedes* larvae. Based on annual surveillance studies during 2016–2020, a tremendously high risk of dengue transmission in the study areas was detected, with an overall HI of 86.1% (95% CI: 83.4–88.4), 20.0% CI (95% CI: 19.4–20.7), and 362 BI (Table 5). Data were calculated to determine the risk of disease transmission in each area. Entomological surveys were conducted in 495 houses (67.6%) in the commercial city and 237 houses (32.4%) in the rural area (Table 5). In the commercial city, the larval indices were 85.5% HI, 20.6% CI, and 387.5 BI, whereas in the rural area, the larval indices were 87.3% HI, 18.7% CI, and 308.9 BI. The average number of containers per house was 19 and 16 in the commercial city and the rural area, respectively. Based on the WHO guidelines, the larval indices determined in both areas revealed a high risk of dengue transmission.

A comparison of the HI and CI values between the commercial city and the rural area is presented in Table 6. According to binary regression analysis, all values obtained from study years 2017 to 2020 were compared with those obtained from study year 2016. The HI values were not significantly different among years, either in the commercial city or the rural area (*p* > 0.05; Table 6). Nevertheless, the CI values showed significant differences among years in both study areas (*p* < 0.05; Table 6). This can imply that *Aedes* larvae habitually occupy households in all locations, while the availability of water-holding containers could be an outcome of vector control measures. The impact of such diverse locations will be further discussed.

#### 2.2.3. Breeding Container Classification 

For the water-holding container classification, the characteristics of the inspected containers were classified using data records during 2017–2020 (Table 7, Table 8 and Table 9). Four categories of water-holding containers, including container usage types, container types, material types, and natural container types were identified. According to the findings, 77.6% of the investigated containers were used in daily routine activities (n = 8845), while the remaining (22.4%) were discarded containers (n = 2546; Table 7A). *Aedes* larvae, on the other hand, were detected in discarded containers at a higher rate than in containers that were used routinely (*p* < 0.05). Our results showed that the discarded containers or trash around houses created significantly more *Aedes* breeding sites than the routinely used water containers. There was an association between the larval presence and the type of container usage (Pearson’s chi-square test: χ^2^ = 165.78, df = 1, *p* < 0.001).

Among the different water container types, pails, buckets, basins, and boxes were the most frequently observed containers (24.5%, n = 2796, Table 7). This container group was mainly utilized in daily routine activities (27.3%, n = 2411, Table 8). Tires created significantly more *Aedes* breeding sites (59.6% larval positive, *p* < 0.05) than other types (Table 7). Other observed container types ranging in order from greatest to least were grouped as follows: vase, cup, bowl, bottle, and can (n = 2184); jar and pot (n = 2007); tank, pond, and cistern (n = 1295); drum and gallon (n = 1003); cover and sheet (n = 467); other containers (n = 513); and dish, plate, saucer, tray, ant trap (n = 380). The natural containers appeared to be the least frequently found containers in the study areas (n = 118). Pearson’s Chi-square test showed that there was an association between larval presence and the type of container (χ^2^ = 930.58, df = 9, *p* < 0.001). 

For the material type category, most inspected water containers were made from plastic (48.7%, n = 5486) (Table 7), with 47 percent of them being utilized in routine activities in the home (Table 8A). Other observed containers were made from clay (23.5%, n = 2610), cement (12.2%, n = 1379), metal (6.7%, n = 751), rubber (6.1%, n = 690), glass (2.8%, n = 313), and other material types (0.4%, n = 44) consisting of paper, stone, wood, and Styrofoam (Table 7). Although rubber was not the most observed material, it significantly contributed to larval production, with 55.2% compared to other material types (*p* < 0.001), followed by cement (23.7%) and clay containers (21.5%; Table 7). Pearson’s Chi-square test showed an association between larval presence and the container material type (χ^2^ = 625.67, df = 6, *p* < 0.001). Mosquito larvae were detected in natural containers (such as coconut shells and plant components), accounting for 11.9% larval infestation in these hidden containers (Table 7). The statistical analysis also showed an association between the natural containers and larval presence (χ^2^ = 17.38, df = 4, *p* < 0.05). 

Table 8 demonstrates that plastic made up over half of the discarded containers (n = 1280), followed by rubber (26.2%, n = 667). The presence of *Aedes* larvae in all discarded containers is presented in Table 9. *Aedes* larval infestations were found in high numbers in used tires (49.5%, n = 374), as well as rubber (50.4%, n = 381) and plastic (33.2%, n = 251) materials.

## 3. Discussion

Since DENV transmission is directly related to its vectors, mosquito-based DENV surveillance in KPP was conducted in areas where dengue cases have been reported. Adult mosquito collections were performed on day 1 and re-performed on day 14 following the case reports. After insecticide application, the number of collected mosquitoes on day 14 was lower than those collected on day 1. Significantly lower numbers of total collected mosquitoes, female *Aedes* vectors, and other species were captured on day 14 compared to day 1. This shows the high effectiveness of the vector control measures and insecticide applications performed by the public health local vector control teams, which immediately reduce the density of target vectors and prevent disease transmission in communities. Although the insecticide space spraying method has been proven to temporarily reduce vector density, its ability to reduce the risk of disease transmission remains unclear [19,20]. 

According to the PCR results collected in this study, the head and thorax of the mosquitoes collected on day 1 were DENV-positive. This implied that they had previously fed on dengue-infected patients for 10–14 days or longer, based on the extrinsic incubation period, prior to feeding on the index case, and then they were captured on the first day of mosquito collection in our study. Moreover, some infected mosquitoes were captured after insecticide application in both index and neighboring houses. This can be explained by the fact that the insecticide’s effectiveness may decline or it may no longer be able to kill existing infected mosquitoes, newly emerging adults, or new mosquito populations that have recently arrived in the area. Consequently, some mosquitoes remain in the houses and can further transmit the disease. 

From our investigations, the 6.44% DENV infection rate in mosquitoes collected in the index houses was significantly higher than 0.8% in neighboring houses. This implies that other family members have a high risk of being exposed to the infectious mosquitoes and becoming infected. The dengue studies in KPP also reported that 8.2% and 9.9% of mosquitoes collected in index houses were DENV-infected, compared to 0.4% in houses without dengue-infected patients [17,21]. The authors emphasized the positive association between DENV infection in mosquitoes and humans at the individual household level and the important role of index cases in transmitting DENV to mosquitoes and to neighbors living nearby. This supports our findings that the infected mosquitoes spread from the index houses to the neighboring houses and can further transmit the disease within the community. The DENV serotype detected in some mosquitoes in this study did not correspond with the index serotype. It is possible that the mosquitoes may have acquired DENV from unreported, asymptomatic infected humans, by vertical transmission [22], or the patient may have acquired DENV from elsewhere. It is also possible that the index cases could have acquired the infection by traveling to neighboring houses, and then infecting the mosquitoes around their home. Supporting results from a spatial dynamics study of DENV transmission in KPP suggested that human movement has a potential role in spreading the pathogen between communities [20], and a consistent result has also been reported regarding the dengue epidemic in Iquitos, Peru [23]. Therefore, not only high vector density in the areas but also human movement plays a vital role in increasing DENV transmission. 

Piped water in many villages in KPP is not available, requiring the storage of water in plastic buckets and earthen jars for daily use, including drinking, cooking, washing, and cleaning. Moreover, villagers prefer to store tap water in cement tanks and clay pots to keep the water cool for bathing. Although, recently, the water system has been improved and made more reliable, the tendency to store water in households remains. Eight water storage jars for routine use were previously found in each house in Thailand [14]. From our observations in the current study, approximately eighteen water-holding containers (both for routine use and discarded containers) were found in each household in KPP. This reflects the cultural tradition of Thai people to keep and use containers to store water for household usage, while those that are no longer used are discarded. These water storage containers are generally found to contain *Aedes* larvae. Many water containers were uncovered because lids and screens are often not properly designed and are not practical for daily use. All of these factors facilitate mosquito breeding and increase the dengue transmission risk. According to the results, the obtained larval indices in this study were extremely high in both areas, indicating the considerably high risk of dengue transmission in these communities. The CI values and the average number of observed containers per household in the commercial city appeared to be higher than those in the rural area. Urbanization and a lack of waste management in the commercial city could explain the numerous routinely used and discarded containers. Lower CI values were detected when the larval survey was conducted in the rural area during study year 2019. At this time period, HI values were also similarly decreased. This could reflect the local vector control campaign activity, as source reduction strategies and larvicide application are clearly required in order to control *Aedes* vector population densities. Therefore, CI and HI could be used as discriminative indicators for dengue epidemics for disease and vector control approaches.

The results of this study identify the major breeding sites of *Aedes* larvae commonly found in KPP. A large number of pails, buckets, basins, boxes, jars, pots, tanks, ponds, cisterns, drums, gallons, and some smaller containers, such as vases, cups, bowls, bottles, and cans, were commonly found for use in routine household activities, and the majority were made from plastic and clay or earthenware. Plastic and earthenware were also reported to be the most frequently observed materials (67%) in the container classification during an immature dengue surveillance study in KPP, Thailand [24]. Additionally, more than 60% of immature stage mosquitoes were detected in plastic containers, which produced approximately 50% of pupae of *Ae. aegypti* in Zanzibar city, Tanzania [25]. In the current study, the remaining discarded containers were also generally made from plastic and rubber. Used tires and other discarded containers, including natural containers, were usually found around residential areas. Although they were less frequently observed when compared to the routinely used containers, our results showed greater *Aedes* larval abundance. Our current findings are consistent with those of a previous study conducted in 2016 in KPP, Thailand, which found a small number of discarded coconut shells around houses and most of them were infested with *Aedes* larvae [26]. This observation is also in agreement with previous studies in other regions of the Central African Republic and the Republic of the Congo, where the lack of pipe water promotes the storage of water in households and there is a lack of waste management [27,28]. Tires, which were the discarded containers that were most commonly infested with larvae, are one of the most important sources of adult vector populations, since they generated 59.6% of the *Aedes* larval presence in our study. They were shipped from place to place by tire-retreading facilities from infested areas and introduced *Aedes* vectors into urban areas [14]. From our observations, the used tires were usually left outside and served as a resting site for adult mosquitoes. They become potential breeding sources when filled with water during the rainy season. Dark rubber materials containing stagnant water are attractive breeding sites for gravid females to lay their eggs. Discarded tires filled with nutritious leaves and organic matter serve as key breeding containers for *Aedes* mosquitoes. Tires were identified as one of the most common container types infested with *Ae. aegypti* larvae or pupae in an entomological survey in KPP [26]. The organic components of sweet waste materials from fruits or vegetables in garbage are also useful for *Aedes* mosquitoes as energy sources and oviposition sites [29]. Larvicide application (e.g., temephos) has been widely encouraged and applied in containers for daily usage. This could decrease the larval density in routinely used containers; however, larvicide application in all discarded containers is impossible. They are often overlooked, despite the fact that they are one of the most important containers that provide a breeding environment for *Ae. aegypti* and *Ae. albopictus*.

In our study, both *Ae. aegypti* and *Ae. albopictus* adults were trapped inside houses, which is consistent with an earlier study that demonstrated larvae of both species coexisting in the same natural containers (coconut shells, husks, and bracts) [30]. Colonization of both vector species was reported to be especially high in trash and discarded containers [27,28,31]. Sharing the same vectors as other arboviruses, e.g., Zika and Chikungunya viruses, may explain this. The invasion and circulation of these arboviruses in the same geographical area as DENV may lead to the rapid spread of disease transmission. Therefore, reducing the numbers of these key containers could lead to a reduction in the dengue vector population and consequently reduce the dengue transmission risk. 

According to this study, larval surveys may provide a relative measure of larval breeding habitat density. Under a field survey, the more households and containers inspected, the more informative indices can be assessed to determine the risk level. However, larval index determination alone is insufficient to define the precise level of transmission in most situations, and the indices are generally not correlated with disease incidence or outbreak risk [32]. The unpredictability of dengue incidence each year, as shown in our study, is another factor that impacts disease transmission, including the number of viremic imports in the area. However, knowledge of the key container types and material types are useful for vector control programs in order to identify the individual container types that produce the most mosquitoes. For example, in this study, while tires comprised only 5.51% of the total number of investigated containers, they accounted for the majority of total *Ae. aegypti* production (59.55%) when compared to other container types. Importantly, the identification of key container types, material types, and container usage types will lead to site-specific and cost-effective control programs if treatment can be focused on the key containers that produce the most adult *Aedes* vectors. 

This study demonstrated a high risk of DENV infection at the individual house level, where humans and mosquitoes are in contact. Entomological surveillance facilitates timely decisions regarding disease control, limiting human–vector exposure, and preventing disease outbreak. Regular monitoring of the abundance of vectors, particularly in areas with positive cases or suspected cases, can be applied. For non-endemic countries where there is a risk of importing dengue cases or vectors, entomological surveillance should be implemented at the focal points of entry, e.g., airports, ports, and ground crossings. Additionally, education about tropical diseases and their vectors should be provided to the operating officer. 

## 4. Materials and Methods

### 4.1. Ethics Statement

This study was approved by the Institutional Review Board of Walter Reed Army Institute of Research (protocol #2119). Written informed consent was obtained from household owners (age ≥ 18 years) in order to enter residences for mosquito surveillance and collection.

### 4.2. Study Sites

**Figure 2:**
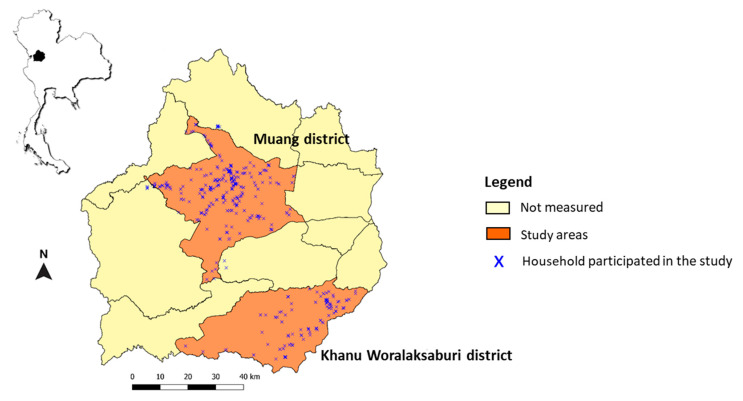
Entomological study sites in Muang and Khanu Worralaksaburi districts, Kamphaeng Phet province, Thailand, during January 2016–August 2020. Each cross (x) represents the collection houses that participated in this study.

Kamphaeng Phet (KPP) is situated in Northern Thailand, with an area of 8600 km^2^. It experiences a tropical climate with marked rainfall seasonality. Several dengue studies have been conducted in this area, providing useful information for continued entomological risk assessment study for DENV transmission. In the current study, the entomological study was performed in 27 dengue-endemic villages located in the Muang and Khanu Woralaksaburi districts from January 2016 to August 2020 (Figure 2). Muang district, the commercial capital of KPP, covers an area of 1349 km^2^ with a population of 215,229. It is divided into 36 local public health offices established for the management of health concerns at the provincial level [33]. The district of Khanu Woralaksaburi has an area of 1159 km^2^ with 112,909 habitants and consists of 18 public health offices.

### 4.3. Entomological Risk Assessment

The entomological risk assessment was divided into two studies (the entomological study in dengue case areas and the annual entomological surveillance). Two mosquito surveillance methods were used in this study: adult collections and larval surveys. Adult collections were performed indoors to collect *Aedes* mosquito vectors, while larval surveys were carried out both indoors and outdoors. Indoors refers to areas covered by a roof, while outdoors refers to outside areas within the vicinity of the residential area.

Adult Mosquito Collection Procedures

Indoor collection was performed using Biogent-Sentinel traps (BG traps) baited with 1 kg of dry ice and BG lure containing ammonia, lactic acid, and caproic acid combinations. Two BG traps were placed inside each surveyed house for approximately 8 h (8 am–4 pm). Upon collection, mosquito-collecting bags were removed, stored in a container filled with dry ice, and transported back to the AFRIMS Entomology Field Laboratory in KPP. The samples were morphologically identified to species level under stereo microscopes, sorted by sex, and counted.

Larval and Container Survey Procedures

All water-holding containers located both indoors and outdoors were examined for the presence of *Aedes* larvae. Water in the containers was poured into a white plastic tray and larvae were collected using disposable clear plastic pipettes. If the containers were too heavy or the water could not be poured out, the water was agitated and the larvae were sampled using a fine net. Torchlights were applied to examine larvae in dark containers such as tires and large cement jars. All observed water-holding containers were grouped and classified for their characteristics into three categories according to the type of container (jar, pot, tank, pond, cistern, vase, cup, bowl, bottle, can, pail, bucket, basin, box, drum, gallon, tire, dish, plate, saucer, tray, ant trap, natural containers, and other containers), the material (clay, plastic, metal, cement, glass, rubber, Styrofoam, paper, wood, and stone), and the container usage type (routine use and discarded containers).

The Larval Indices

The larval indices were implemented to measure dengue vector infestation in this study, i.e., house index (HI, proportion of *Aedes*-positive houses), container index (CI, proportion of *Aedes*-positive containers), and Breteau index (BI, number of *Aedes*-positive containers per 100 houses) [2]. These three measures are currently the most commonly used indices to assess dengue vector larval breeding habitat infestations. The indices were calculated in accordance with the WHO guidelines. A high risk of dengue transmission is determined when HI > 10% and BI > 50, and low transmission risk is determined when HI < 1% and BI < 5 [34]. All collected data from both studies were used to determine the risk of transmission in the study areas. 

#### 4.3.1. Entomological Study in Dengue Case Areas

This study was initiated in order to measure entomological risk factors in dengue transmission areas. All confirmed dengue cases were diagnosed by the PCR technique, and the local health centers were informed by the Kamphaeng Phet AFRIMS Virology Research Unit (KAVRU) team. The entomological surveillance, including adult mosquito collections and larval surveys in houses with confirmed, active dengue cases, were conducted by entomologists from the AFRIMS Entomology Department within 24 h (day 1) of receiving the dengue-positive case reports. The coordinates of each collection house were collected and mapped. A total of 35 dengue cases were reported from January 2016 to August 2020. All 51 houses (35 family units, comprising 35 houses containing dengue cases (index houses) and 16 houses of their neighbors that were located in the same area) were accessed to perform the entomological surveillance. The adult mosquito collections were re-performed in the same 51 houses on day 14 after each case report. In this study, a total of 102 visits were performed. Based on the Thai MOPH vector management standard protocol, mosquito vector control (both adulticide and larvicide applications) was conducted within 24 h of each case report, which was done after mosquito sampling. Our results on day 14 after case reports can be used to determine the efficacy of insecticide spraying during the disease transmission period.

Sample Preparation and Nested RT-PCR for DENV Detection in Mosquitoes

For DENV detection in the vector samples, females of *Ae. aegypti* and *Ae. albopictus* were individually dissected on a chilled table. To detect DENV infection in the salivary glands, which have high potential for DENV transmission, the head and thorax parts of each female were separated from the abdomen and stored in a single 1.5 mL microcentrifuge safe-lock tube filled with 200 µL RPMI media, supplemented with 10% fetal bovine serum, 1% Pen/Strep, and a silver grinding bead. The samples were ground with the Bullet Blender^®^ Storm24 (Next Advance, Inc., Troy, NY, USA) for 2 min at a speed of 6 and stored on wet ice for transportation to the Virology department for further DENV detection using the quantitative real-time PCR (RT-PCR) technique. Viral RNA was extracted from 140 μL of mosquito suspension using the QIAamp viral RNA mini kit (QIAGEN, Hilden, Germany) according to the manufacturer’s instructions and AFRIMS SOP. Nested RT-PCR was conducted to detect and identify the DENV type following the method previously described [35]. The assay includes two steps: the first step of RT-PCR uses DENV universal primers and the second step is a nested PCR using DENV-type-specific primers. The amplification was performed on the Mastercycler^®^ nexus (Eppendorf, Hamburg, Germany) following the manufacturer’s instructions. The PCR products were analyzed using gel electrophoresis. Each DENV type was identified based on the specific size of its PCR products. 

#### 4.3.2. Annual Entomological Surveillance Study

The annual entomological surveillance was initiated in order to determine the infestation of adult mosquito vectors and potential breeding habitats, and to evaluate transmission risks in disease-endemic areas. The surveillance was annually conducted during the dengue transmission period (May to October) from 2016 to 2020 in households participating in this research study (n = 801) in Muang district (commercial city) and Khanu Woralaksaburi district (rural area). Adult collection in each house was typically carried out in a single day. A total of 1002 trap-days were performed in 501 randomly surveyed houses to collect adult mosquitoes. Larval survey and container investigation were conducted in 732 houses in the same area during the same period as adult collection. 

### 4.4. Statistical Analysis

All statistical analyses were performed using IBM SPSS version 25. For the entomological study in dengue case areas, the numbers of mosquitoes collected on day 1 and 14 following the dengue case reports were compared. Because data were not normally distributed, Wilcoxon signed rank test (nonparametric test of paired *t*-test) was used to compare variables. Fisher’s exact test was performed to determine the DENV infection rate in *Aedes* vectors on different collection dates and analyze the relationships among study houses (index and neighboring houses). Mann–Whitney U test was used to compare the numbers of collected mosquitoes between houses with and without DENV-positive mosquito samples. This statistical method was also applied for the association analysis between larval presence and study houses with or without DENV-positive mosquitoes. For the annual entomological surveillance, a binary logistic regression model was used to assess the relationship between variables (study area and study year) and larval presence in study households (HI) and in observed containers (CI). Odds ratios (OR) and their 95% confidence intervals (95% CI) were estimated. The characteristics of the observed containers, including the type of container usage, container type (both artificial and natural containers), material type, and natural container types, were analyzed for their relationship with the presence of *Aedes* larvae using Pearson’s Chi-square test. The proportion z-test was performed to determine levels of significant differences among container characteristics. The dataset for the larval and container survey in study year 2016 was excluded from calculation because it lacked some details, which may have affected the interpretation of the results.

## 5. Conclusions

The positive detection of DENV in *Ae. aegypti* mosquitoes collected from houses associated with dengue cases has significant relevance for public health and vector control measures in DENV transmission areas. This study revealed that the DENV infection rate in *Ae. aegypti* mosquitoes was 4.9%. Most infected mosquitoes were captured in the index houses on the first day after a case was reported, and others were collected on day 14 after insecticide application. This highlights the ineffectiveness of insecticide spraying, since a single application of non-residual insecticide was not sufficient to diminish the DENV transmission risk. An integrated vector management concept including chemical control, environmental management, source reduction, and waste management should be emphasized and encouraged among the villagers in communities. In this study, the villagers stored water in different container types and placed them inside and around their residential areas. Containers were kept replenished with pipe water and rain water all year round, which enabled the vectors to breed. While household water containers are common, this study identified used tires and other discarded containers as major breeding habitats of dengue vectors. The larval indices (house index, container index, and Breteau index) were sufficiently high to represent a risk of *Aedes* vector-borne diseases. It is clear that there is a high risk of potential disease transmission in these areas. Targeting specific types of water-holding containers would help to eliminate all unnecessary and discarded containers. Since a good mosquito vector surveillance program is much less expensive than a control program, our research suggests that vector surveillance should be conducted regularly to provide information to public health authorities so that they can design effective vector control plans for disease prevention and effectively assess the risk of dengue transmission. The integration of different methods should also be taken into consideration. 

## Figures and Tables

**Table 1:** Adult mosquito collections on day 1 and day 14 in entomological study in dengue case areas.

Adult Mosquito Collection	Day 1	Day 14	Total	*p*-Value
Dengue case	35	35		
Index houses (n)	35	35	70	
Neighbor houses (n)	16	16	32	
Total inspected houses (n)	51	51	102	
House with the presence of *Aedes* * female vectors (n)	44	40	84	
BG traps (n)	102	102	204	
Total collected mosquitoes (n)	741 ^a^	475 ^b^	1216	0.002
Total collected *Aedes* * female vectors (n)	311 ^a^	181 ^b^	492	0.002
Average of *Aedes* * female vectors per trap	3	2	2	
Other collected mosquitoes ** (n)	430 ^a^	294 ^b^	724	0.019
PCR-tested mosquito samples (n)	286	165	451	
DENV-positive mosquito samples (n)	19	3	22	
Mosquito infection rate (%)	6.64 ^a^	1.82 ^b^	4.9	0.023
**Index houses**				
PCR-tested mosquito samples (n)	210	116	326	
DENV-positive mosquito samples (n)	18	3	21	
Mosquito infection rate (%)	8.57 ^a^	2.57 ^b^	6.44	0.036
**Neighboring houses**				
PCR-tested mosquito samples (n)	76	49	125	
DENV-positive mosquito samples (n)	1	0	1	
Mosquito infection rate (%)	1.3	0	0.8	
DENV serotypes (No. positive mosquito samples)	DENV-1 (6)	DENV-3 (2)		
	DENV-2 (5)	DENV-4 (1)		
	DENV-3 (4)			
	DENV-4 (4)			

* Both *Ae. aegypti* and *Ae. albopictus* females; ** *Aedes* male, *Culex* sp., *Anopheles* sp., *Mansonia* sp., *Armigeres* sp., Different lowercase letters indicate differences at *p*-value < 0.05.

**Table 2:** Comparison of DENV infection rates in *Aedes* vectors collected from index and neighboring households.

Study Households	House Inspected (n)	PCR-Tested Mosquito Samples (n)		DENV Infection Rate in Mosquito Samples (%)	χ^2^	df	*p*-Value
		Pos^+ve^	Neg^+ve^				
**Index houses**	70	21	305	6.44 ^a^	6.198	1	0.012
**Neighboring houses**	32	1	124	0.8 ^b^			
**Total**	**102**	**22**	**429**				

Mosquito collections were performed in 35 index houses and 16 neighboring houses on day 1 and day 14; Pos^+ve^: Positive; Neg^+ve^: Negative. Different lowercase letters indicate differences at *p*-value < 0.05.

**Table 3:** Association analysis between larval-positive containers and study households.

	Study Households	House Inspected (n)	Container Inspected (n)	Larval-Pos^+ve^ Container (%)	χ^2^	df	*p*-Value
A	Index houses	35	573	18.67 ^a^	6.38	1	0.012
	Neighboring houses	16	227	11.01 ^b^			
	**Total**	**51**	**800**				
B	Houses with DENV-Pos^+ve^ mosquitoes	12	248	26.61 ^a^	25.63	1	<0.001
	Houses with DENV-Neg^+ve^ mosquitoes	39	552	11.96 ^b^			
	**Total**	**51**	**800**				

Larval and container investigations were performed in 35 index houses and 16 neighboring houses on day 1; Pos^+ve^: Positive; Neg^+ve^: Negative. Different lowercase letters indicate differences at *p*-value < 0.05.

**Table 4:** Comparisons of adult mosquito collection among study years in Muang district (commercial city areas) and Khanu Woralaksaburi district (rural areas).

	Study Area	2016	2017	2018	2019	2020	Total
**A**	**All study areas**						
	Houses inspected (n)	71	96	90	104	140	501
	BG trap (n)	142	192	180	208	280	1002
	Total mosquitoes (Mean ± SE)	1519 (21.39 ± 3.91)	1901 (19.80 ± 2.11)	1158 (12.87 ± 2.19)	1809 (17.39 ± 2.48)	533 (3.81 ± 0.53)	6920 (13.81 ± 1.00)
	*Aedes* * female vectors (Mean ± SE)	687 (9.68 ± 2.20)	1166 (12.15 ± 1.38)	647 (7.19 ± 1.36)	932 (8.96 ± 1.47)	221 (1.58 ± 0.21)	3653 (7.29 ± 0.59)
	*Aedes* * female vectors (%)	45.23	61.34	55.87	51.52	41.46	52.79
**B**	**Muang district (commercial city)**						
	Houses inspected (n)	71	71	68	65	103	378
	BG trap (n)	142	142	136	130	206	756
	Total mosquitoes (Mean ± SE)	1519 (21.39 ± 3.91)	1250 (17.61 ± 2.17)	697 (10.25 ± 1.84)	1418 (21.82 ± 3.66)	220 (2.14 ± 0.28)	5104 (13.50 ± 1.17)
	*Aedes* * female vectors (Mean ± SE)	687 (9.68 ± 2.20)	791 (11.14 ± 3.91)	402 (5.91 ± 1.28)	701 (10.78 ± 2.13)	109 (1.06 ± 0.14)	2690 (7.12 ± 0.69)
	*Aedes* * female vectors (%)	45.23	63.28	57.68	49.44	49.55	52.70
**C**	**Khanu** **Woralaksaburi district (rural area)**						
	Houses inspected (n)	0	25	22	39	37	123
	BG trap (n)	NA	50	44	78	74	246
	Total mosquitoes (Mean ± SE)	NA	651 (26.04 ± 5.14)	461 (20.95 ± 6.76)	391 (10.03 ± 2.09)	313 (8.46 ± 1.63)	1816 (14.76 ± 1.88)
	*Aedes* * female vectors (Mean ± SE)	NA	375 (15.00 ± 3.30)	245 (11.14 ± 3.85)	231 (5.92 ± 1.61)	112 (3.03 ± 0.62)	963 (7.83 ± 1.16)
	*Aedes* * female vectors (%)	NA	57.60	53.15	59.08	35.78	53.03

* Both *Ae. aegypti* and *Ae. albopictus* females; NA: not applicable.

**Table 5:** Entomological indices of *Aedes* vectors according to the study areas: all study areas (A), Muang district—commercial city (B), and Khanu Woralaksaburi district—rural area (C).

	Study Year	House Inspected (n)	House with *Aedes* Larvae (n)	Container Inspected (n)	Container with *Aedes* Larvae (n)	Container per House (n)	Larval Indices		
							HI (95% CI)	CI (95% CI)	BI
A	All study areas								
	**2016**	**108**	**89**	1835	305	17	82.4 (74.4–88.7)	16.6 (15.0–18.4)	282.4
	2017	107	97	1922	505	18	90.7 (84.1–95.1)	26.3 (24.3–28.3)	472.0
	2018	136	124	2769	626	20	91.2 (85.5–95.1)	22.6 (21.1–24.2)	460.3
	2019	167	132	2798	430	17	79.0 (72.4–84.7)	15.4 (14.1–16.7)	257.5
	2020	214	188	3904	784	18	87.9 (83.0–91.7)	20.1 (18.8–21.4)	366.4
	**Total**	**732**	**630**	**13,228**	**2650**	**18**	**86.1 (83.4–88.4)**	**20.0 (19.4–20.7)**	**362.0**
B	Muang district (commercial city)								
	2016	85	68	1449	234	17	80.0 (70.6–87.4)	16.1 (16.1–18.1)	275.3
	2017	73	66	1371	366	19	90.4 (82.1–95.6)	26.7 (24.4–29.1)	501.4
	2018	79	71	1650	419	21	89.9 (81.8–95.1)	25.4 (23.3–27.5	530.4
	2019	94	77	1717	290	18	81.9 (73.2–88.7)	16.9 (15.2–18.7)	308.5
	2020	164	141	3136	609	19	86.0 (80.0–90.6)	19.4 (18.1–20.8)	371.3
	**Total**	**495**	**423**	**9323**	**1918**	**19**	**85.5 (82.1–88.4)**	**20.6 (19.8–21.4)**	**387.5**
C	Khanu Woralaksaburi district (rural area)								
	2016	23	21	386	71	17	91.3 (74.9–98.1)	18.4 (14.8–22.5)	308.7
	2017	34	31	551	139	16	91.2 (78.3–97.5)	25.2 (21.7–19.0)	408.8
	2018	57	53	1119	207	20	93.0 (84.2–97.6)	18.5 (16.3–20.9)	363.2
	2019	73	55	1081	140	15	75.3 (64.6–84.1)	13.0 (11.0–15.1)	191.8
	2020	50	47	768	175	15	94.0 (84.8–98.3)	22.8 (19.9–25.9)	350.0
	**Total**	**237**	**207**	**3905**	**732**	**16**	**87.3 (82.7–91.1)**	**18.7 (17.5–20.0)**	**308.9**

HI: House index; CI: Container index; BI: Breteau index; 95% CI: 95% confidence interval.

**Table 6:** Logistic regression results on factors influencing the presence of *Aedes* larvae in study households and in observed water-holding containers.

	Variable	House Index			Container Index		
		OR	95% CI	*p*-Value	OR	95% CI	*p*-Value
**A**	**All study areas**						
	Commercial city: Rural area	1.227	0.766–1.968	0.395	0.886	0.804–0.976	0.014
	2017	2.030	0.894–4.606	0.090	1.805	1.538–2.117	<0.001
	2018	2.119	0.974–4.611	0.058	1.500	1.287–1.748	<0.001
	2019	0.770	0.410–1.443	0.414	0.930	0.792–1.093	0.379
	2020	1.538	0.808–2.927	0.190	1.259	1.088–1.456	0.002
	Constant	4.494		<0.001	0.204		<0.001
**B**	**Muang district (commercial city)**						
	2017	2.357	0.918–6.053	0.075	1.891	1.573–2.273	<0.001
	2018	2.219	0.899–5.478	0.084	1.767	1.478–2.113	<0.001
	2019	1.132	0.536–2.391	0.744	1.055	0.874–1.274	0.576
	2020	1.533	0.768–3.057	0.226	1.251	1.060–1.477	0.008
	Constant	4.000		<0.001	0.193		<0.001
**C**	**Khanu** **Woralaksaburi district (rural area)**						
	2017	0.984	0.151–6.404	0.987	1.497	1.085–2.064	0.014
	2018	1.262	0.215–7.416	0.797	1.007	0.747–1.357	0.963
	2019	0.291	0.062–1.364	0.117	0.660	0.483–0.902	0.009
	2020	1.492	0.232–9.601	0.674	1.309	0.962–1.781	0.086
	Constant	10.500		0.001	0.225		<0.001

OR: odds ratio; 95% CI: 95% confidence interval; Ref: the reference study area is commercial city and the reference study year is 2016.

**Table 7:** Classification of water-holding containers: container usage types (A), container types (B), material types (C), and natural container types (D).

	Category	Container Classification	Container Inspected (%)	Pos^+ve^ Container (%)	χ^2^	df	*p*-Value
**A**	**Container usage types**						
		Routine use container	8845 (77.6)	1589 (18.0) ^a^	165.78	1	<0.001
		Discarded container	2546 (22.4)	756 (29.7) ^b^			
		**Total container inspected**	**11,391**	**2345 (20.6)**			
**B**	**Container types**						
		Jar/pot	2007 (17.6)	512 (25.5) ^a^	930.58	9	<0.001
		Tank/pond/cistern	1295 (11.4)	311 (24.0) ^a,b^			
		Vase/cup/bowl/bottle/can	2184 (19.2)	193 (8.8) ^c^			
		Pail/bucket/basin/box	2796 (24.5)	426 (15.2) ^d^			
		Drum/gallon	1003 (8.8)	258 (25.7) ^a^			
		Tire	628 (5.5)	374 (59.6) ^e^			
		Dish/plate/saucer/tray/ant trap	380 (3.3)	119 (31.3) ^a^			
		Cover/sheet	467 (4.1)	60 (12.8) ^d^			
		Natural containers	118 (1.0)	14 (11.9) ^b,c,d^			
		Other containers *	513 (4.5)	78 (15. 2) ^d^			
		**Total container inspected**	**11,391**	**2345 (20.6)**			
**C**	**Material types**						
		Clay	2610 (23.5)	561 (21.5) ^a^	625.67	6	<0.001
		Plastic	5486 (48.7)	929 (16.9) ^b^			
		Metal	751 (6.7)	111 (14.8) ^b^			
		Cement	1379 (12.2)	327 (23.7) ^a^			
		Glass	313 (2.8)	13 (4.2) ^c^			
		Rubber	690 (6.1)	381 (55.2) ^d^			
		Other materials **	44 (0.4)	9 (20.5) ^a,b^			
		**Total container inspected**	**11,273**	**2331 (20.7)**			
**D**	**Natural container types**						
		Coconut shell	87 (73.7)	8 (9.2) ^a^	17.38	4	0.005
		Bamboo stump	5 (4.2)	2 (40.0) ^a,b^			
		Tree hole	5 (4.2)	3 (60.0) ^b^			
		Snail shell	7 (5.9)	1 (14.3) ^a,b^			
		Plant parts	14 (11.9)	0 (0.0) ^a^			
		**Total container inspected**	**118**	**14 (11.9)**			

Pos^+ve^ = Positive; Different lowercase letters indicate differences at *p*-value < 0.05. * Other containers: appliances, bath tub, boat, helmet, plowshare, ice breaker, cellphone case, umbrella. ** Other materials: paper, stone, wood, Styrofoam.

**Table 8:** Classification of routine use containers (A) and discarded containers (B) and their materials as observed in annual entomological surveillance study during 2017–2020.

	Container Usage Type	Clay (%)	Plastic (%)	Metal (%)	Cement (%)	Glass (%)	Rubber (%)	Others * (%)	Total (%)
**A**	**Routine use containers**								
	Jar/Pot	1726	30	39	147	1			**1943 (22.0)**
	Tank/Pond/Cistern	32	67	7	1133	21			**1260 (14.2)**
	Vase/Cup/Bowl/Bottle/Can	689	551	95	51	236		1	**1623 (18.3)**
	Pail/Bucket /Basin/Box	28	2280	87	14			2	**2411 (27.3)**
	Drum/Gallon	1	875	25					**901 (10.2)**
	Dish/Plate/Saucer/Tray/Ant trap	35	207	31		1			**274 (3.1)**
	Cover/Sheet	3	148	20					**171 (1.9)**
	Others **	5	48	177	7		23	2	**262 (3.0)**
	**Total**	**2519** **(28.5)**	**4206** **(47.6)**	**481** **(5.4)**	**1352** **(15.3)**	**259** **(2.9)**	**23** **(0.3)**	**5** **(0.1)**	**8845**
**B**	**Discarded containers**								
	Jar/Pot	54	1	5	4				**64 (2.5)**
	Tank/Pond/Cistern		9	5	19	2			**35 (1.4)**
	Vase/Cup/Bowl/Bottle/Can	14	390	90	1	48		18	**561 (22.0)**
	Pail /Bucket/Basin/Box		316	56				13	**385 (15.1)**
	Drum/Gallon		95	7					**102 (4.0)**
	Tire						628		**628 (24.7)**
	Dish/Plate/Saucer/Tray/Ant trap	4	79	19	1	1		2	**106 (4.2)**
	Cover/Sheet		275	21					**296 (11.6)**
	Natural container								**118 (4.6)**
	Others **	19	115	67	2	3	39	6	**251 (9.9)**
	**Total**	**91** **(3.6)**	**1280** **(50.3)**	**270** **(10.6)**	**27** **(1.1)**	**54** **(2.1)**	**667** **(26.2)**	**39** **(1.5)**	**2546**

* Other materials: paper, stone, wood, Styrofoam; ** Other containers: appliances, bath tub, boat, helmet, plowshare, ice breaker, cellphone case, umbrella.

**Table 9:** Number of discarded containers with the presence of *Aedes* larvae observed in annual entomological surveillance study during 2017–2020.

Positive Discarded Containers	Clay (%)	Plastic (%)	Metal (%)	Cement (%)	Glass (%)	Rubber (%)	Others * (%)	Total (%)
Jar/Pot	17		2	1				**20 (2.6)**
Tank/Pond/Cistern		3	2	11	1			**17 (2.2)**
Vase/Cup/Bowl/Bottle/Can	2	47	21	1	5		3	**79 (10.4)**
Pail /Bucket/Basin/Box		90	13				2	**105 (13.9)**
Drum/Gallon		33						**33 (4.4)**
Tire						374		**374 (49.5)**
Dish/Plate/Saucer/Tray/Ant trap		20	7					**27 (3.6)**
Cover/Sheet		35	2					**37 (4.9)**
Others **	3	23	13		1	7	3	**50 (6.6)**
Natural container	NA	NA	NA	NA	NA	NA	NA	**14 (1.9)**
**Total**	**22** **(2.9)**	**251** **(33.2)**	**60** **(7.9)**	**13** **(1.7)**	**7** **(0.9)**	**381** **(50.4)**	**8** **(1.1)**	**756**

* Other materials: paper, stone, wood, Styrofoam; ** Other discarded containers: appliances, bath tub, boat, helmet, plowshare, ice breaker, cellphone case, umbrella.

## Data Availability

Not applicable.

## Supplementary Materials

The following are available online at https://www.mdpi.com/article/10.3390/pathogens10101234/s1, Table S1: Number of positive *Ae. aegypti* samples collected on day 1 and day 14 and DENV infection rates during the study in 2016–2020.
