# Supplementary material for: Entomological Risk Assessment for Dengue Virus Transmission during 2016–2020 in Kamphaeng Phet, Thailand
Source: Pathogens. 2021 Sep 24;10(10):1234. doi: 10.3390/pathogens10101234 (PMC8538081; doi:10.3390/pathogens10101234)
Supplement: Supplementary file 1 [file pathogens-10-01234-s001.zip › pathogens-1322921-supplementary.pdf]

Supplementary Materials

Table S1. Number of positive *Ae. aegypti* samples collected on day 1 and day 14 and DENV infection rates during the study in 2016-2020.

Study Year	Month	Index Case No.	DENV Serotype in index	DENV Serotype in Mosquitoes								DENV Pos+ve Mosquitoes (% Infection)
				Day 1				Day 14				
				DENV-1	DENV-2	DENV-3	DENV-4	DENV-1	DENV-2	DENV-3	DENV-4	
**2016**	**JAN**	**1**	DENV-4									
	MAR	2	DENV-4									
	JUL	3	DENV-4									
	AUG	4	DENV-4				1					**1**
**2017**	JUN	5	DENV-2		3							**3**
	JUN	6	DENV-4									
	JUN	7	DENV-4									
	JUN	8	DENV-2									
	AUG	9	DENV-4				1					**1**
	NOV	10	DENV-3			3				2		**5**
	DEC	11	DENV-1									
**2018**	FEB	12	DENV-1	1								**1**
	MAY	13	DENV-2		2							**2**
	JUN	14	DENV-4									
	JUN	15	DENV-1	2								**2**
	JUN	16	DENV-1									
	JUN	17	DENV-1									
	JUN	18	DENV-1, DENV-3			1						**1**
	JUL	19	DENV-1									
	JUL	20	DENV-3									
	JUL	21	DENV-1									
	JUL	22	DENV-3									
	OCT	23	DENV-2									
	NOV	24	DENV-1									
	NOV	25	DENV-2									
	NOV	26	DENV-3									
	DEC	27	DENV-1									
**2019**	JAN	28	DENV-4									
	MAR	29	DENV-1	1								**1**
	JUN	30	DENV-4				1					**1**
	JUL	31	DENV-2									
	JUL	32	DENV-2									
	AUG	33	DENV-2									
**2020**	JUL	34	DENV-1								1	**1**
	JUL	35	DENV-1	2			1					**3**
**Total**		**35**		**6**	**5**	**4**	**4**	**0**	**0**	**2**	**1**	**22 (4.9)**

Pos+ve = Positiv.e
